# Unraveling symptom interplay: a network analysis of procrastination in gifted students

**DOI:** 10.1186/s40359-024-01868-6

**Published:** 2024-06-28

**Authors:** Sajjad Bagheri, Hojjatollah Farahani, Peter Watson, Timea Bezdan, Kosar Rezaiean

**Affiliations:** 1 Clinical Psychology, Department of Psychology, Hakim-Toos Institute of Higher Education, Mashhad, Iran; 2 Department of Psychology, Faculty of Humanities, Tarbiat Modares University, Tehran, Iran; 3 MRC Cognition and Brain Sciences Unit, University of Cambridge, Cambridge, UK; 4 Faculty of Informatics and Computing, Singidunum University, Belgrade, Serbia; 5 MA Candidate in Clinical Psychology, University of Tehran, Tehran, Iran

**Keywords:** Symptom interplay, Network analysis, Procrastination, Rumination, Perfectionism, Cognitive flexibility, Gifted adolescents, Psychological well-being

## Abstract

**Background:**

This study explores the intricate web of symptoms experienced by academically gifted high school students, focusing on procrastination, rumination, perfectionism, and cognitive flexibility. The well-being of these gifted adolescents remains a pivotal concern, and understanding the dynamics of these symptoms is vital.

**Methods:**

A diverse sample of 207 academically gifted high school students from Mashhad, Iran, participated in this study. Using convenience sampling, participants from grades 10, 11, and 12 were included, with detailed assessments conducted through questionnaires measuring the mentioned symptoms.

**Results:**

Our network analysis uncovers compelling insights into the interplay of these symptoms: Procrastination, though moderately central, exerts significant influence within the network, underscoring its relevance. Cognitive flexibility, while centrally positioned, curiously exhibits a negative influence, potentially serving as a protective factor. Negative perfectionism emerges as the keystone symptom, with both high centrality and a positive influence. Rumination displays substantial centrality and a positive influence, indicating its role in symptom exacerbation. Positive perfectionism, moderately central, lacks direct influence on other symptoms.

**Conclusion:**

This network analysis provides a nuanced understanding of the relationships among procrastination, rumination, perfectionism, and cognitive flexibility in academically gifted adolescents. Negative perfectionism and cognitive flexibility emerge as critical factors deserving attention in interventions aimed at enhancing the well-being of this unique group. Further research should explore causal relationships to refine targeted interventions.

## Introduction

The identification of gifted students has long perplexed educators, giving rise to the Elitism movement in many developed countries [1]. In the modern world, formal education is designed to be inclusive, offering opportunities from kindergarten through higher education to individuals of all genders, ethnic backgrounds, and social statuses [2]. Within this educational framework, some students exhibit typical academic performance, with their efforts aligning with a median level of achievement. Conversely, there exists a distinct group of students whose exceptional abilities set them apart, consistently outshining their peers of the same age [3]. These academically gifted students often receive early recognition as high achievers, a distinction that holds promise for their future success [4]. However, the concept of giftedness remains complex and can be subject to misconceptions within school systems [5]. Gifted students display superior performance across various domains of development compared to their peers [6]. Yet, their heightened sensitivity and perfectionist tendencies, spanning mental, psychomotor, emotional, and sensory dimensions, can sometimes hinder their personal communication and social interactions [7, 8]. These gifted individuals, often characterized by unique thinking patterns, a penchant for questioning, and the ability to provide innovative solutions, hold great importance for the advancement of societies [9]. Notably, talent, manifesting in mental, musical, artistic, physical, and social realms, often accompanies high intelligence levels [10].

In their pursuit of excellence, gifted individuals may exhibit behaviors such as withdrawal, giving up, or disengaging from their environment if they believe they cannot attain the desired results [11]. Loneliness can also be a consequence of their unique experiences, as evidenced by Ünal and Sak’s (2020) study titled " The extraordinary ones: Lonely adolescents with giftedness” which highlighted the reactions of gifted students in educational settings and the potential loneliness resulting from their distinctiveness [12]. The research demonstrated that gifted students frequently face reactions such as jealousy and exclusion from their peers due to their distinctive thinking, advanced skills, and higher levels of success and talent. In the pursuit of competence in achievement-oriented environments, some gifted students implicitly internalize self-worth and strive to be the best [13]. This pursuit of excellence is often intertwined with perfectionism, a personality trait that has been associated with various adaptive and maladaptive outcomes in academically gifted youth [13, 14]. Perfectionism, recognized as a multidimensional construct, is categorized into normal perfectionism, characterized by the pursuit of high standards and excellence, and neurotic perfectionism, which results in anxiety and dissatisfaction [15, 16]. Procrastination, defined as the nonadaptive behavior of involuntarily postponing planned tasks without a clear reason [17], has been linked to adverse academic performance and negative emotional outcomes, including depression, anxiety, and shame, among college students [18–22]. The relationship between perfectionism and procrastination has been the subject of investigation, with specific facets of perfectionism predicting procrastination tendencies [23, 24].

Rumination, a stable personality trait involving repetitive and passive contemplation of the causes and potential consequences of negative life events, has also been associated with perfectionism and depressive symptoms [25, 26]. Rumination is a process wherein individuals with high levels of this trait tend to dwell on negative experiences rather than engage in constructive coping strategies [27]. Cognitive flexibility, on the other hand, is the ability to adapt one’s thinking and behavior to changing environmental demands [28]. High levels of cognitive flexibility enable individuals to navigate challenging situations, generate alternative ideas, and employ problem-solving skills effectively [29]. The interplay among perfectionism, procrastination, rumination, and cognitive flexibility in gifted adolescents represents a complex psychological landscape. To explore these intricate relationships, network analysis offers a novel approach. Network analysis allows for the examination of the interactions and central symptoms within this unique population, shedding light on the structure of psychological disturbances and potential intervention targets [30–34]. In this study, we aim to contribute to a deeper understanding of these psychological dynamics and provide insights for interventions that promote the well-being and academic success of gifted adolescents.

Network analysis has emerged as a novel approach to conceptualizing psychological phenomena in a manner that addresses the limitations of the traditional approach. In network theory, central symptoms are more likely to activate other symptoms and play a major role in causing the onset and/or maintenance of a syndrome/disorder. Network analysis has the potential to map specific relationships among individual symptoms of a disorder and identify targets for treatment [35]. Furthermore, network analysis can be used to extract the structure of psychiatric disturbances from clinical data [31] and highlight meaningful associations between individual symptoms within and/or between disorders [36]. Additionally, a network model is useful in understanding the mechanism of comorbidities and provides suggested strategies for clinicians to prevent and treat comorbidities [34]. Researchers have used network analysis to assess these symptom-symptom interactions. Network analysis, a set of procedures based on the modeling of dynamical systems [37], provides a visual depiction of the complex associations among symptoms. A tightly connected network with many strong connections among symptoms is considered a ‘riskier’ network because activation of one symptom can quickly spread to other symptoms, leading to more chronic symptoms over time [38]. Network analysis also allows the identification of highly ‘central’ or influential symptoms, defined by having, on average, strong connections with other symptoms. When a highly central symptom is activated (i.e. a person reports the presence of the symptom), it will influence other symptoms to become activated as well, maintaining the symptom network. To date, most network studies have examined symptom relationships and centrality within a single disorder. However, network analysis may be particularly useful for understanding co-morbidity because it permits the identification of potential pathways from one disorder to another [39].

## Methods

In this section, we present a detailed account of the methodology employed in our study. The research design was carefully crafted to address key research questions and objectives, employing a combination of quantitative measures and advanced analytical techniques. The following subsections outline the participant selection process, measurement tools utilized, and the analytical framework applied for data interpretation.

### Participants and study design

In this comprehensive study, the target population comprised high school students with outstanding talents at the secondary level in the city of Mashhad. The participants were exclusively enrolled in Hashemi Nejad High School, an institution under the auspices of the National Organization for the Development of Exceptional Talents of Iran. Admission to this school is contingent upon successfully passing specialized entrance tests assessing both intelligence and academic aptitude. To determine the sample size, the Morgan table was employed, considering a total population of 450 gifted male students in Mashhad. The target population was calculated to encompass 207 students from the second-grade secondary school at Hashemi Nejad High School during the academic year 2022–2023. Convenience sampling was chosen as the method due to the accessibility of the sample. The process of filling out the questionnaire and the required time commitment were clearly explained to all participants to ensure uniformity in data collection. It is noteworthy that the participants in this study represent a diverse group of academically gifted high school boys, encompassing various ethnic backgrounds and socio-economic statuses. This diversity enhances the generalizability of the findings to a broader population of academically talented high school students.

### Measures

To gather data and information for analysis and hypothesis testing in the research, the following questionnaires will be employed:

#### Positive and negative perfectionism scale (PANPS)

This scale was developed by Terry-Short et al. (1995) and consists of two sub-scales, each comprising 20 questions [40]. These sub-scales assess positive and negative perfectionism, with each sub-scale containing 20 questions. Responses to these questions are based on a 5-point Likert scale ranging from 1) strongly disagree to 5) strongly agree, resulting in scores ranging from 20 to 100. Scoring for this questionnaire is structured in such a way that if an individual scores high on questions related to positive perfectionism, they are categorized as having a positive perfectionism orientation, while a high score on negative perfectionism indicates an orientation towards negative perfectionism. The cutoff score for individuals displaying negative perfectionism tendencies is 69 or higher.

#### Cognitive flexibility inventory (CFI)

Developed by Dennis and Vander Wal in 2010, this questionnaire comprises 20 seven-point Likert scale questions, with response options ranging from 1 (strongly disagree) to 7 (strongly agree) [28]. It measures cognitive flexibility, with higher scores indicating greater cognitive flexibility. The questionnaire demonstrates good validity, with a correlation of 0.75 with the Beck Depression Inventory [41]. In Iran, Shahre et al., (2014) reported a Cronbach’s alpha of 0.71 for the entire scale [41].

#### Rumination response scale (RRS)

This 22-item scale, developed by Nolen-Hoeksema, is rated on a 4-point scale from 1 to 4 and assesses cognitive distortions. The Cronbach’s alpha of this scale has been reported to range from 0.88 to 0.92, indicating high internal consistency [42]. Intra-class correlation for the 5 retest measurements was 0.75, and the test-retest reliability over more than 12 months was 0.67. In Iran, Bagherinejad et al. (2010) reported correlations of 0.79 with depression and 0.56 with anxiety [43].

#### Tuckman procrastination scale

For assessing procrastination tendencies, the standard Tuckman Procrastination Scale (1991) is employed [44]. This self-report scale consists of 16 items, rated on a Likert scale. Higher scores on this scale indicate higher levels of procrastination. Tuckman (1991) reported a reliability coefficient of 0.86 for this scale. In Kazemi et al.‘s (2010) research, Cronbach’s alpha for the entire scale was 0.71, indicating good reliability [45].

### Statistical analysis

The data utilized in this study underwent a comprehensive multi-step analytical approach, integrating symptom network analysis and correlation stability analysis. The primary objectives of the research were as follows:

#### Aim 1: Characterization of the symptom network at admission

##### Edges calculation

The initial phase involved computing polychoric correlations [46] between all items in the dataset. Polychoric correlations, selected due to the ordinal nature of the variables, were employed to estimate associations assumed to be continuous and normally distributed. The resulting correlation matrix served as input for constructing the symptom network. The network, modeled using a Graphical Gaussian Model (GGM), represented conditional independence relationships between nodes. To ensure a concise network, the graphical lasso (glasso) algorithm was applied, effectively shrinking smaller edges to zero [47–49].

##### Network visualization

The resultant symptom network underwent visualization using the qgraph R package. The edge thickness in the visualization indicated the strength of associations, while the Fruchterman-Reingold algorithm determined the spatial arrangement of nodes. Nodes with stronger average associations were strategically positioned closer to the center of the network [50, 51].

##### Centrality measures

Node centrality was assessed through three key indices: strength (sum of absolute edge weights connected to a node), closeness (average distance to all other nodes), and betweenness (number of times a node lies on the shortest path between two other nodes). These centrality measures provided valuable insights into the relative importance of individual symptoms within the network [36, 52].

#### Aim 2: Stability assessment of the symptom network

##### Network stability

Network stability was rigorously evaluated using a permutation-based method. The dataset underwent random division into multiple sub-samples, and independent networks were estimated from each subsample. Edge and centrality values were then correlated across these independent networks. This process was iterated 10,000 times to comprehensively assess the stability of the symptom network [53].

##### Confidence intervals

Confidence intervals (CIs) for edge values were calculated using a bootstrap approach [38]. Additionally, the stability of centrality values was examined by repeatedly correlating values derived from the complete dataset with those obtained from subsamples with varying percentages of nodes or participants missing [54].

#### Aim 3: Comparison between admission and discharge networks

##### Global network strength

Changes in the global network strength between admission and discharge were systematically assessed using the Network Comparison Test (NCT). A null distribution was created through random swapping of participants’ admission and discharge data, constructing networks, and computing NCT scores over 10,000 iterations [55].

##### Network structure

Alterations in network structure were evaluated by correlating edge values and centrality indices between admission and discharge networks. The magnitude of these correlations provided valuable insights into the stability of the network structure over time.

All data analyses were executed using the R statistical programming language, with relevant packages and functions employed for network estimation, inference, stability assessment, and regularization.

## Results

### Demographic findings

The distribution of ages in our sample is presented in Table 1. The majority (88.5%) of the high school students are aged 16 or 17.

**Table 1:** Descriptive information

Age	Frequency	Percentage
15	5	2.5%
16	86	41.4%
17	98	47.1%
18	17	8.3%
19	1	0.6%
total	207	100%

### Network analysis and correlation stability

In this section, we present an analysis of the network structure and centrality measures based on the provided data. The analysis encompasses various aspects of the network, including node centrality, edge betweenness, shortest path lengths, and correlation stability.

**Fig. 1:**
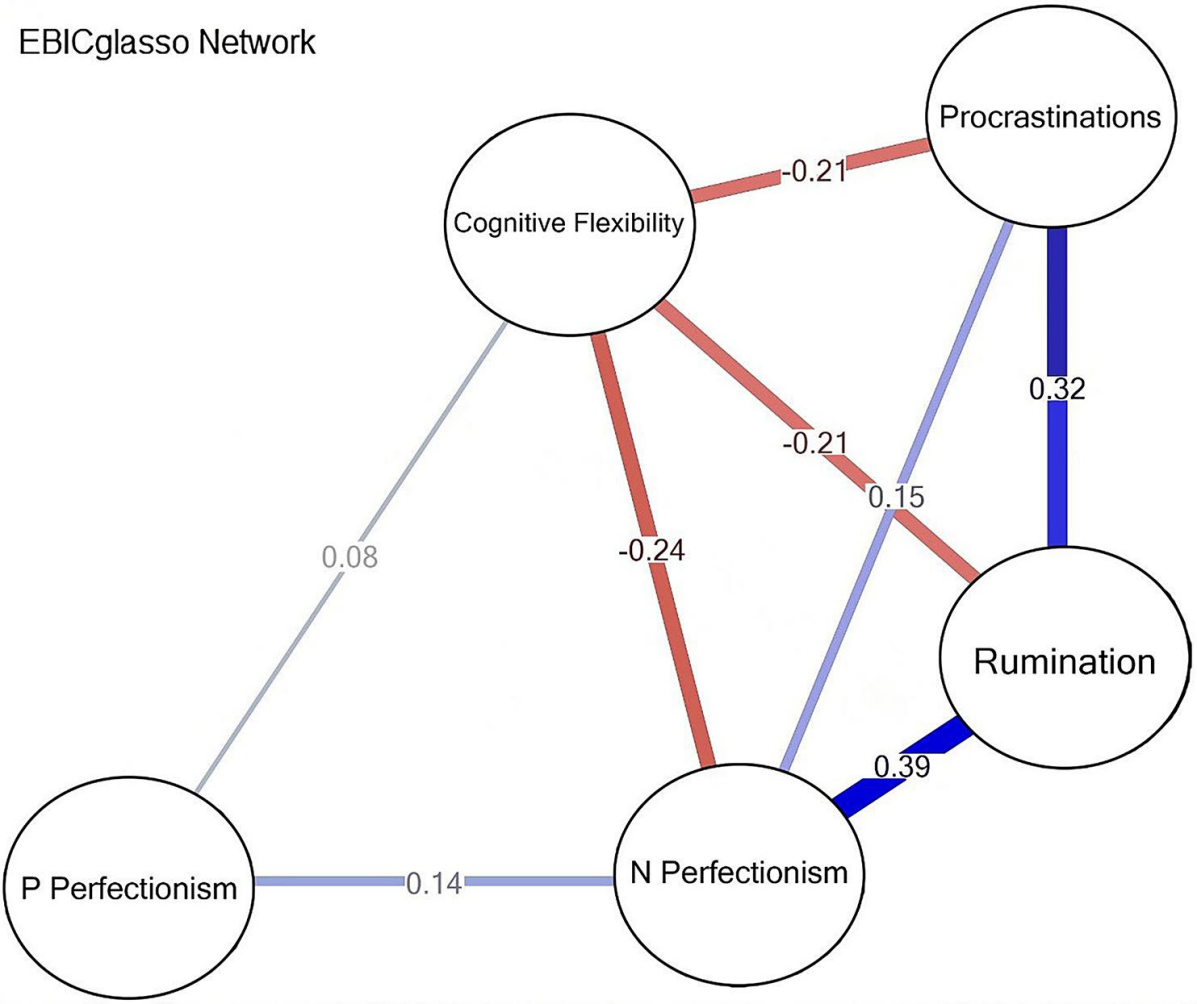
Network structure of procrastination, perfectionism, rumination and cognitive flexibility in gifted students

Figure 1 estimated network model for procrastination, perfectionism, rumination and cognitive flexibility in gifted students. Notably, the depiction includes visual elements to enhance understanding. Nodes with stronger connections are depicted closer to each other in the diagram, reflecting the intensity of their relationships. Red lines indicate negative correlations, while blue lines represent positive correlations. The thickness of the edges corresponds to the strength of the association between symptom nodes. This visual representation aids in comprehending the intricate relationships and dynamics within the network of procrastination, perfectionism, rumination, and cognitive flexibility among gifted students.

### Node centrality measures

The network consists of several nodes representing different variables. Node centrality measures, such as betweenness (looking at how many short paths between nodes feature the node of interest), closeness (how influential a node is in indirect connections to all other nodes), and strength (how influential a node is in direct connections to all other nodes), provide insights into the importance of each node within the network. These measures are crucial for understanding the flow of information or influence within the network and are further described below.

Here are some key findings regarding node centrality:

- Procrastinations has a closeness centrality of 0.0376 and a strength centrality of 0.6773.
- Cognitive Flexibility exhibits a closeness centrality of 0.0398 and a strength centrality of 0.7380.
- Negative Perfectionism shows a closeness centrality of 0.0510 and a strength centrality of 0.9234.
- Rumination has a closeness centrality of 0.0495 and a strength centrality of 0.9144.
- Positive Perfectionism has a closeness centrality of 0.0244 and a strength centrality of 0.2228.

**Fig. 2:**
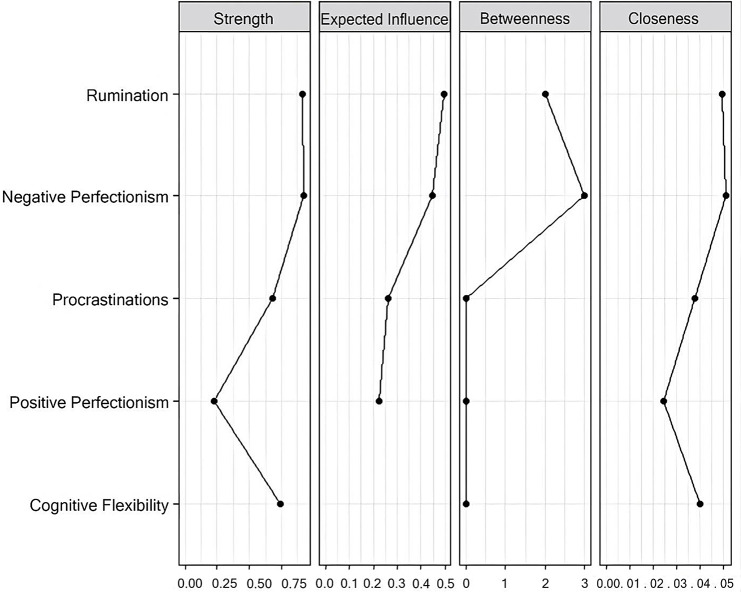
Centrality indices of network nodes based on z scores

These centrality measures highlight the nodes’ relative importance within the network and suggest all the above variables except Positive Perfectionism have direct connections between one another (Fig. 2).

### Edge betweenness

Edge betweenness measures the number of pairs of nodes whose shortest path includes the edge which runs between a specified pair of nodes. For example:

- There are four pairs of nodes whose shortest path includes the edge between Negative Perfectionism and Rumination, indicating their relationship represents a relatively strong central connection in the network acting as a bridge between other nodes.
- Procrastinations and Cognitive Flexibility have a direct relationship since only one shortest path includes the edge between them.

These edge betweenness values provide insights into the flow of influence or information in the network that runs between specific pairs of nodes.

**Fig. 3:**
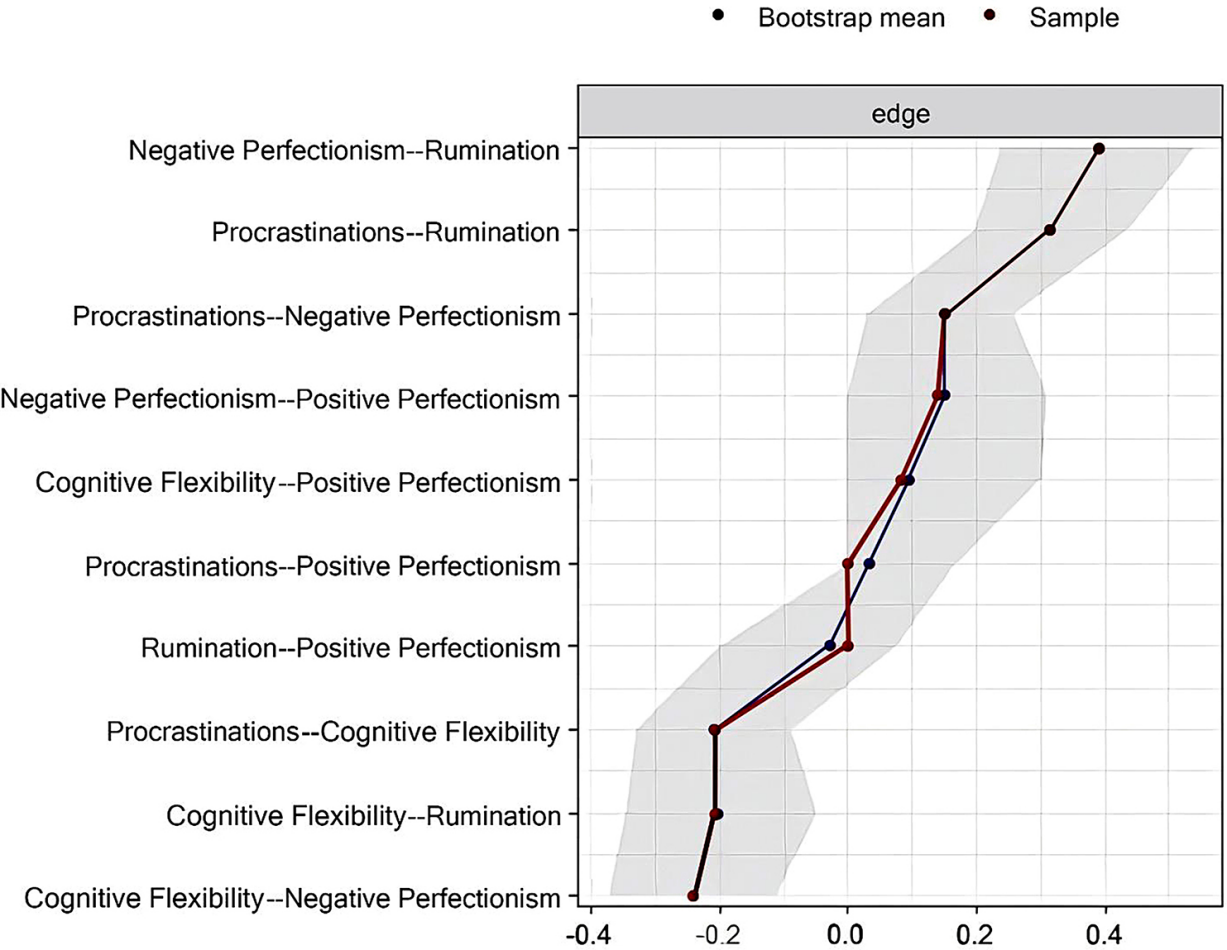
Bootstrap results to determine 95% confidence intervals for the edge weight test

Please refer to Fig. 3 for the bootstrap results, which determine the 95% confidence intervals for the edge weight test.

**Fig. 4:**
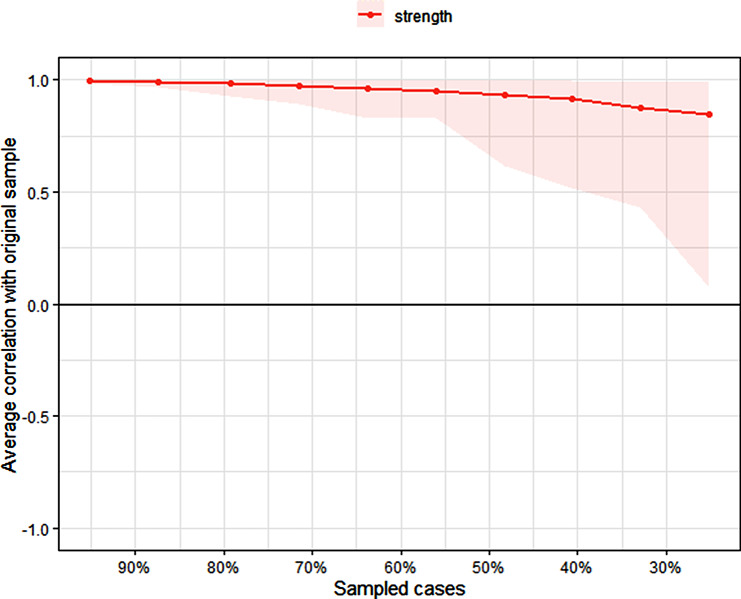
Stability of centrality indices by case dropping subset bootstrap. The x-axis represents the percentage of cases of the original sample used at each step. The y-axis represents the average of correlations between the centrality indices in the original network and the centrality indices from the re-estimated networks after excluding increasing percentages of cases. The line indicates the correlations between bridge strengths in the reduced and original samples.

In Fig. 3, we present the bootstrap results to determine the 95% confidence intervals for the edge weight test. These confidence intervals provide an indication of the precision of our estimates, representing a range of values within which we are reasonably confident that the true population parameter lies. The edge weights in our analysis signify the strength of associations between different symptom nodes in the network. Through bootstrap resampling, we generate multiple samples from our original data to estimate the variability in the edge weights, allowing us to quantify the uncertainty surrounding our estimates. A narrower confidence interval suggests a more precise estimate of the edge weight, indicating less variability across different samples. Conversely, a wider confidence interval suggests greater uncertainty and less precision in our estimates. Therefore, by presenting the 95% confidence intervals for the edge weights in Fig. 3, we provide readers with a measure of the precision of our estimates and the degree of certainty surrounding the strength of associations between symptom nodes in the network.

### Shortest path lengths

The shortest path lengths represent the minimum number of edges that must be traversed to move from one node to another. Closeness and betweenness both depend on the concept of shortest path lengths. The shortest path length between two given nodes refers to the shortest distance between these two nodes based on the edges that directly or indirectly connect these two nodes. Dijkstra’s algorithm is used to find shortest path lengths in weighted networks [55–57]. Based on this algorithm, shortest path lengths represent the inverse of edge weights that have to be “travelled” on the shortest path. Closeness sums the shortest path lengths between a given node and all other nodes in the network and takes the inverse of the resulting value. Therefore, closeness represents how likely it is that information from a given node “travels” through the whole network either directly or indirectly. Betweenness represents how strongly a given node can disrupt information flow in the network, as betweenness calculates the number of shortest paths a given node lies on. For instance:

- The shortest path length between Procrastinations and Cognitive Flexibility is approximately 4.8163. This value is obtained by determining the shortest path between these two nodes, accounting for the weights of the edges traversed along the path.
- Between Cognitive Flexibility and Negative Perfectionism, the shortest path length is around 4.1795. Similarly, this value is calculated based on the shortest path between these nodes within the network structure.
- Negative Perfectionism and Procrastinations have a shortest path length of approximately 5.7302. Again, this value is computed by finding the shortest path between these nodes while considering the network topology and edge weights.

These path lengths provide valuable insights into the proximity and accessibility between nodes in the network, offering a quantitative measure of the distance between symptom nodes. By employing established algorithms for calculating shortest paths, we ensure robustness and accuracy in our analyses, allowing for a comprehensive understanding of the network structure.

### Correlation stability analysis

In network analysis, ensuring the stability of the results is paramount to establish the reliability of the findings. To address this, we employed the Correlation Stability (CS) coefficient as a pivotal measure. The CS coefficient serves to quantify the stability of the network by evaluating the consistency of correlations across various subsets of the data, indicating the extent to which the network structure remains intact when parts of the sample are excluded. CS-C values represented the maximum proportion of samples that could be removed. Generally, a CS-C above 0.50 is preferred, ensuring a robust network structure [56]. Key findings include:

- The strength of the network exhibits excellent stability, with a CS coefficient of 0.517. This value indicates that approximately 51% of the sample could be dropped without substantially affecting the network’s overall structure.
- Moreover, it’s important to note that this CS coefficient is associated with a 95% confidence interval, providing a measure of certainty around the reported stability metric.
- Additionally, bootstrap analyses were conducted to further confirm the reliability and stability of the estimated edge weights. These analyses involved resampling the data to assess the variability in the edge weights and ensure robustness in our findings.

The significance of stability analysis lies in its ability to reassure readers about the robustness of the network findings, demonstrating that they are not unduly influenced by specific data points (Fig 4). By reporting the CS coefficient alongside its associated confidence interval, we offer a comprehensive assessment of the network’s stability, instilling greater confidence in the reliability of our results.

### Network comparison tests

Comparative analyses were conducted to evaluate potential differences in network models based on demographic factors. These tests aimed to identify variations in network strength and edge weights between different groups, such as gender, school grade, and residence [57].

#### Key findings include

- No significant differences in network global strength were observed between male and female adolescents.
- Some specific edge weights showed differences between genders, suggesting variations in symptom associations.
- Subdividing the sample by school grade or residence did not reveal significant differences in network global strength or edge weight distribution.

These network comparison tests provide insights into how demographic factors may influence the network structure and symptom associations. However, it is important to note that due to the relatively small sample size, the network comparison tests might not fully capture the demographic influences. The limited sample size restricts the statistical power necessary for a robust comparison, potentially resulting in non-positive definite correlation matrices. This limitation should be considered when interpreting the results, and future research with larger samples is recommended to more definitively explore these demographic influences.

In summary, network analysis and correlation stability assessment offer valuable insights into the relationships between variables, the importance of specific nodes, and the stability of the network findings. Additionally, while network comparison tests suggest potential demographic influences on symptom associations, the current sample size limits the ability to draw definitive conclusions. Future studies with larger samples are needed to further explore these demographic influences and validate the preliminary findings of this study.

## Discussion

The network analysis conducted in this study unveiled intriguing insights into the symptom interplay among academically gifted adolescents.

Procrastination, a pervasive issue among high-achieving students, emerged as a symptom of moderate centrality within the network. This finding aligns with previous research highlighting the prevalence of procrastination among academically gifted individuals [47–50]. However, our study extends this understanding by demonstrating its central role in shaping the symptom network, emphasizing the need for targeted interventions tailored to this population.

Similarly, cognitive flexibility, although centrally positioned, exhibited an unexpected negative influence on the network. This contrasts with conventional views of cognitive flexibility as a positive trait and suggests its potential role as a protective factor among academically gifted students [50–52]. This nuanced understanding challenges traditional assumptions and underscores the importance of considering context-specific factors in intervention development.

The standout revelation of our analysis was the dominance of negative perfectionism, characterized by both high centrality and a positive influence. While previous research has acknowledged the prevalence of perfectionism among gifted students, our study elucidates its pivotal role in shaping symptom dynamics [54, 55,–57]. This underscores the urgency of addressing negative perfectionism in interventions aimed at promoting the well-being of academically gifted adolescents.

Furthermore, rumination, another prevalent symptom in this group, displayed substantial centrality and positive influence, highlighting its contribution to symptom development [58–62]. Our findings complement existing literature on the detrimental effects of rumination and emphasize the need for targeted interventions addressing this symptom among academically gifted individuals.

To translate these findings into practical applications, educators and mental health professionals working with academically gifted students should consider interventions that specifically address procrastination, negative perfectionism, and rumination. Strategies focusing on enhancing cognitive flexibility may serve as a protective factor against the co-occurrence of these symptoms. For instance, incorporating mindfulness practices or cognitive-behavioral interventions tailored to the unique characteristics of gifted students could be explored. Additionally, educators could implement time management and goal-setting techniques to mitigate procrastination tendencies. These practical implications emphasize the importance of tailored interventions to support the well-being of academically gifted adolescents.

The analysis of shortest path lengths provided insights into how quickly information or influence can spread within the network. Notably, procrastination, cognitive flexibility, and negative perfectionism had relatively long shortest path lengths to other symptoms, indicating their centrality. This suggests that these symptoms may act as central nodes in the network, influencing the overall dynamics [63–68].

In contrast, rumination had shorter shortest path lengths, suggesting that it may play a key role in the rapid dissemination of influence or information within the symptom network. This underscores the urgency of addressing rumination in interventions designed for academically gifted adolescents [69, 70].

The correlation stability analysis revealed the robustness of the network’s correlations across various sampling levels. Even with substantial drop percentages, the network’s correlations remained relatively stable, supporting the validity of the network structure and relationships. This finding enhances our confidence in the identified symptom network and its implications for intervention development.

Examining edge betweenness and strength values provided further insights into the network’s dynamics. The high edge value between Negative Perfectionism and Rumination suggests that these symptoms act as bridges, connecting other symptoms in the network. This highlights the potential importance of these two symptoms in mediating the interactions among other symptoms and suggests that interventions targeting them may have broader effects on the symptom network [71–75].

In light of these findings, interventions aimed at enhancing the psychological well-being of academically gifted adolescents should prioritize addressing negative perfectionism, procrastination, and rumination.

Additionally, interventions should consider leveraging the potential protective role of cognitive flexibility [76–79]. Future research should delve deeper into the causal relationships between these symptoms and explore the development of targeted interventions that take into account the complex network dynamics uncovered in this study.

## Conclusion

First and foremost, the centrality measures of the symptoms in the network reveal important patterns. Procrastination, while not having the highest centrality, emerges as a significant factor, underlining the need for targeted interventions to address this behavior among gifted students. On the contrary, cognitive flexibility, with its notable centrality and negative influence, acts as a potential protective factor, deterring the co-occurrence of other symptoms. Understanding the role of cognitive flexibility in this context is crucial for developing interventions that harness its positive impact. Negative perfectionism, identified as a keystone symptom, exhibits the highest centrality and a positive influence on other symptoms. This highlights the critical role of negative perfectionism in shaping the symptom landscape among academically talented students, suggesting that interventions targeting negative perfectionism could significantly contribute to the well-being of this population. Rumination, with its substantial centrality and positive influence, is closely tied to other symptoms, emphasizing its potential to exacerbate or contribute to the development of additional negative symptoms. Recognizing the role of rumination is essential for designing interventions that effectively address this specific aspect. In contrast, positive perfectionism, while moderately central, lacks direct influence on other symptoms. This finding suggests that interventions may need to prioritize mitigating the negative aspects of perfectionism rather than promoting its positive aspects.

The practical implications of addressing procrastination, negative perfectionism, and rumination, along with the recognition of the protective role of cognitive flexibility, highlight the importance of tailored interventions for this unique population. This study contributes significantly to the field by offering insights that can guide educators, mental health professionals, and researchers in supporting the psychological well-being and academic success of academically gifted students. As we continue to explore the dynamics and causal relationships among these symptoms, the findings pave the way for more targeted interventions that address the specific needs of this talented student population, ultimately enriching their academic journey and overall well-being.

In conclusion, this network analysis has shed light on the symptom interplay among academically gifted adolescents, emphasizing the significance of addressing procrastination, negative perfectionism, and rumination while considering the potential role of cognitive flexibility. Building on these insights, future research could explore the causal relationships between these symptoms to better understand the mechanisms driving their interconnected dynamics. For instance, investigating whether high levels of negative perfectionism contribute to increased procrastination tendencies or exploring how cognitive flexibility influences the development of other symptoms could provide valuable insights. By delving into these potential causal links, researchers can refine targeted interventions and further contribute to our understanding of the psychological well-being of academically gifted students.

### Limitations

#### Sample size

One significant limitation of this study is the sample size, which may not be sufficient for extensive network comparison tests across multiple demographic variables. The small sample size could lead to non-positive definite correlation matrices, limiting the reliability of the network comparison findings. Future research should aim to include larger and more diverse samples to enhance the robustness of network comparison tests and better understand the potential demographic influences on symptom associations.

#### Sample characteristics

The study primarily focused on academically gifted high school students from a single school in Mashhad, Iran. The limited geographical and institutional scope may restrict the generalizability of findings to a broader population of academically gifted adolescents. Future research should consider diverse samples from multiple schools and regions to enhance the external validity of the results.

#### Cross-sectional design

The study utilized a cross-sectional design, capturing a snapshot of the symptoms and their relationships at a specific point in time. Longitudinal studies could provide a more dynamic understanding of how these symptoms evolve over time and allow for the exploration of causal relationships between them.

#### Self-report measures

The data relied on self-report measures, which may be subject to response biases, including social desirability or recall bias. Future research could incorporate multi-method assessments, including observational or interview-based measures, to provide a more comprehensive understanding of the studied constructs.

#### Cultural specificity

The study focused on academically gifted students in a specific cultural context (Iran). Cultural factors may influence the expression and perception of symptoms. Future research should consider cultural variations to determine the generalizability of the findings across diverse cultural settings.

#### Intervention implications

While the study provides insights into potential intervention targets, the effectiveness of specific interventions was not directly assessed. Future research should incorporate intervention studies to evaluate the impact of targeted strategies on reducing symptoms and enhancing the well-being of academically gifted adolescents.

### Suggestions for future research

#### Longitudinal investigations

Conduct longitudinal studies to track the development and interaction of symptoms over time. This approach would allow for a more nuanced understanding of causality and changes in symptom dynamics, providing valuable insights for targeted interventions.

#### Diverse cultural samples

Extend the research to include academically gifted students from diverse cultural backgrounds. Examining how cultural factors influence symptom patterns and relationships can contribute to a more comprehensive understanding of the experiences of gifted adolescents.

#### Comparative studies

Undertake comparative studies to explore potential variations in symptom networks among academically gifted students and their non-gifted peers. Understanding the unique challenges faced by gifted students in comparison to their peers can inform tailored interventions.

#### Intervention studies

Implement and evaluate targeted interventions based on the identified symptom network. Assess the effectiveness of interventions designed to address procrastination, negative perfectionism, and rumination, while promoting cognitive flexibility, in improving the well-being of academically gifted adolescents.

#### Qualitative approaches

Complement quantitative findings with qualitative approaches to gain a deeper understanding of the subjective experiences and contextual factors influencing the symptoms. Qualitative data can provide rich insights into the lived experiences of academically gifted students.

#### Incorporate multimodal assessments

Expand assessment methods to include multimodal approaches, such as neurobiological measures or behavioral observations, to triangulate findings and enhance the robustness of symptom characterization.

#### Parental and teacher perspectives

Investigate the perspectives of parents and teachers regarding the observed symptoms in academically gifted students. Understanding how external stakeholders perceive and interact with these symptoms can provide a more holistic view of the challenges faced by gifted adolescents.

## Data Availability

Data is available from the corresponding authors upon request.
